# ATP Synthase *β*-Chain Overexpression in SR-BI Knockout Mice Increases HDL Uptake and Reduces Plasma HDL Level

**DOI:** 10.1155/2014/356432

**Published:** 2014-07-10

**Authors:** Kexiu Song, Yingchun Han, Linhua Zhang, Guoqing Liu, Peng Yang, Xiaoyun Cheng, Le Bu, Hui Sheng, Shen Qu

**Affiliations:** ^1^Department of Endocrinology, Shanghai Tenth People's Hospital, Tongji University, 301 Middle Yanchang Road, Shanghai 200072, China; ^2^Institute of Cardiovascular Science, Key Laboratory of Molecular Cardiovascular Sciences, Ministry of Education, Peking University Health Science Center, Beijing 100191, China; ^3^Nanjing Medical University, Nanjing 210029, China

## Abstract

HDL cholesterol is known to be inversely correlated with cardiovascular disease due to its diverse antiatherogenic functions. SR-BI mediates the selective uptake of HDL-C. SR-BI knockout diminishes but does not completely block the transport of HDL; other receptors may be involved. Ectopic ATP synthase *β*-chain in hepatocytes has been previously characterized as an apoA-I receptor, triggering HDL internalization. This study was undertaken to identify the overexpression of ectopic ATP synthase *β*-chain on DIL-HDL uptake in primary hepatocytes in vitro and on plasma HDL levels in SR-BI knockout mice. Human ATP synthase *β*-chain cDNA was delivered to the mouse liver by adenovirus and GFP adenovirus as control. The adenovirus-mediated overexpression of *β*-chain was identified at both mRNA and protein levels on mice liver and validated by its increasing of DiL-HDL uptake in primary hepatocytes. In response to hepatic overexpression of *β*-chain, plasma HDL-C levels and cholesterol were reduced in SR-BI knockout mice, compared with the control. The present data suggest that ATP synthase *β*-chain can serve as the endocytic receptor of HDL, and its overexpression can reduce plasma HDL-C.

## 1. Introduction

Both epidemiological and clinical studies have demonstrated that the serum levels of high-density lipoprotein (HDL) cholesterol are inversely correlated with the risk of atherosclerosis [1–3]. HDL protects against atherosclerosis and cardiovascular disease by mediating reverse cholesterol transport, protecting vascular endothelium, and exerting antioxidant, anti-inflammatory, and antithrombotic effects [4, 5].

In humans, a 1 mg/dL (0.03 mmol/L) increase in baseline HDL is associated with a 6% decrease in the risk of death from coronary disease [6]. Furthermore, clinical trials have shown that HDL could be an important therapeutic target [7]. Therapy with the HDL mimetic apoAI phospholipid may result in regression of atherosclerosis [7], and these mimetic peptides may also influence the vascular biology of the vessel wall and protect against other acute and chronic inflammatory diseases [8]. Functional integrity of HDL is equally important for its antiatherogenic properties, as one study showed that patients with normal or elevated but functionally abnormal HDL suffered from atherosclerosis [9].

HDL takes up and transports surplus cholesterol from the peripheral tissues to the liver for disposal in bile [10], a process mediated by the HDL cell surface receptors on hepatocytes. Two receptor types have been identified, one is a high affinity receptor—scavenger receptor class B type I (SR-BI) [11] and the other is an endoreceptor—ATP synthase *β*-chain (ATPase-B1). SR-BI binds to HDL with a high affinity and may mediate selective cholesterol uptake of HDL in hepatocytes. SR-BI overexpression may significantly change plasma HDL levels, possibly reducing the incidence of arteriosclerosis [12–16]. Furthermore, SR-BI-knockout models show an increased rate of arteriosclerosis [17–19]. SR-BI is relatively nonspecific, as it binds to LDL, oxidized LDL, and very low density lipoprotein in addition to HDL [20]. ATPase-B1 is also involved in HDL catabolism, as it triggers HDL and apoAI internalization in hepatocytes [21].

ATP synthase is an enzymatic complex (about 600 kDa) responsible for ATP synthesis in mitochondria, prokaryote membranes, and chloroplasts. Mitochondrial ATP synthase is composed of two domains: an extramembranous catalytic domain (F1) and a transmembrane domain (F0) that functions as a proton channel [22]. The mammalian ATP synthase consists of at least 16 different subunits: F1: *α*, *β*, *γ*, *δ*, and *ε*+IF1; F0: a–g, F6, A6L, and andoligomycin-sensitivity conferring protein [23]. It has also been found on the cell surface of endothelial cells, adipocytes, hepatocytes, and tumor cells using immunofluorescence or cell surface biotinylation techniques [24–28] and may be involved in neovascularization, hypertension, cell proliferation, and cytotoxicity [24–30], although the mechanism leading to its ectopic expression is still unknown.

The F1 domain of ATPase contains the catalytic site for ATP synthesis and hydrolysis and the binding sites for ATP and ADP [23]. In endothelial cells, apoA-I binding to ATPase-B1 causes ATP synthase to hydrolyze ATP into ADP and inorganic phosphate. ADP then stimulates apoA-I and HDL uptake into the cell and transendothelial transport of initially lipid-free apoA-I and HDL via activation of the P2Y12 receptor [31].

In the present study, we investigated whether overexpression of ATPase-B1 increases DiI-HDL uptake in primary hepatocytes of wild-type (WT) or SR-BI-knockout (SR-BI−/−) mice. We also sought to determine the effect of exogenously added ATPase-B1 cDNA on plasma HDL-C in SR-BI−/− mice.

## 2. Results

### 2.1. Construction and Amplification of Ad-ATPase-B1

**Figure 1:**
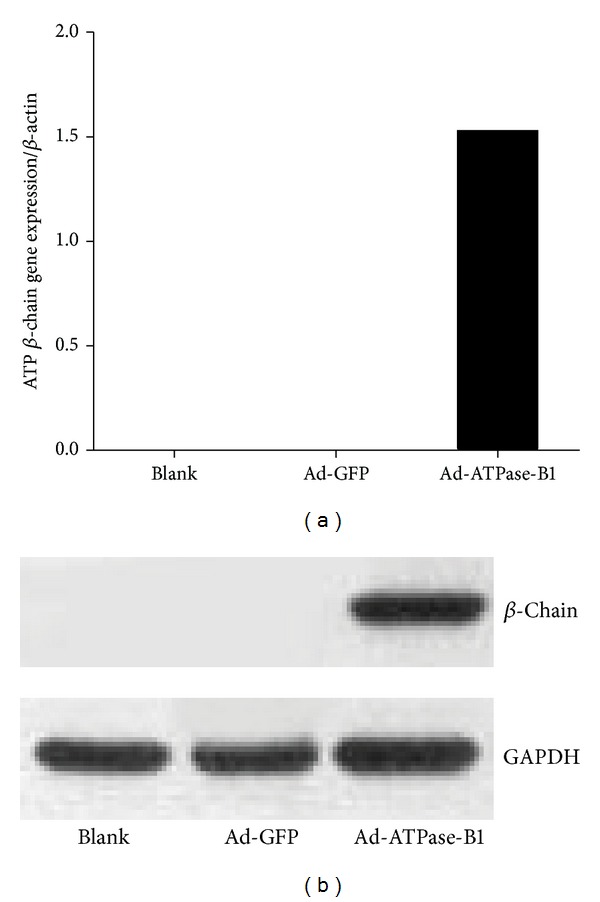
Amplification and identification of Ad-ATPase-B1 in HEK293A cells. Ad-GFP: adenovirus GFP; Ad-ATPase-B1: recombinant adenovirus ATP synthase *β*-chain. (a) Exogenous ATPase-B1 mRNA level in HEK293A cells with no vector (BLANK), pretreated with Ad-GFP and pretreated with Ad-ATPase-B1 and (b) ATPase-B1 protein detected with anti-V5-tag antibody (1 : 2000), GAPDH (1 : 1000).

To further determine the role of ATPase-B1 in HDL metabolism, we transferred human ATPase-B1 cDNA into an adenovirus vector, with a V5-tag on the carboxy terminal end for detection. Increased expression of ATPase-B1mRNA (Figure 1(a)) and protein (Figure 1(b)) was confirmed in HEK293A cells infected with Ad-ATPase-B1 compared with the control (Ad-GFP) vector.

### 2.2. ATPase-B1 Expression in Primary Hepatocytes

**Figure 2:**
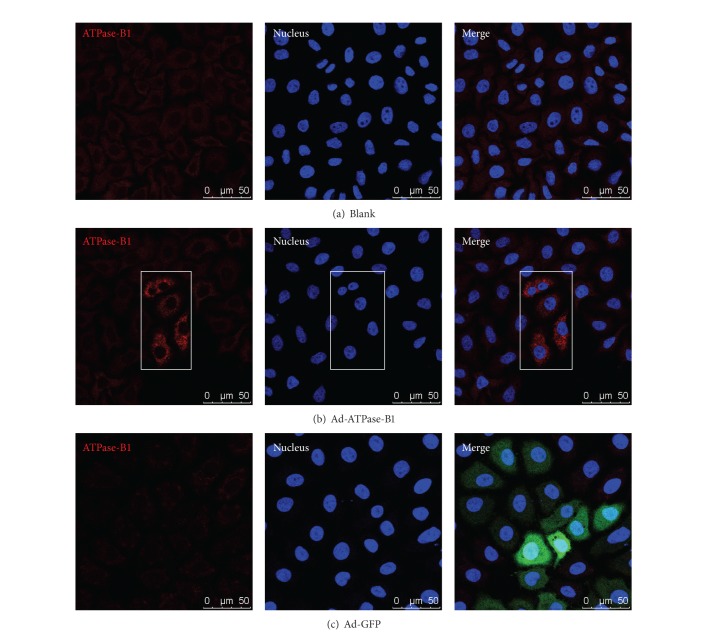
Immunofluorescent confocal microscopic analysis of ATPase-B1 expression in primary hepatocytes. Freshly isolated primary hepatocytes grown on cover slips were pretreated with Ad-ATPase-B1 (b) and Ad-GFP (c) at 30 MOIs for 48 h. After washing with PBS, the cells were immunostained with antibody against V5-tag and AlexaFluor 596-conjugated goat anti-mouse IgG and analyzed by confocal microscope.

To study whether ATPase-B1 affects HDL uptake, we first confirmed its overexpression in primary hepatocytes. Primary hepatocytes infected with Ad-ATPase-B1 showed significantly higher levels of immunodetectable ATPase-B1 expression compared to the control vector (Figure 2).

**Figure 3:**
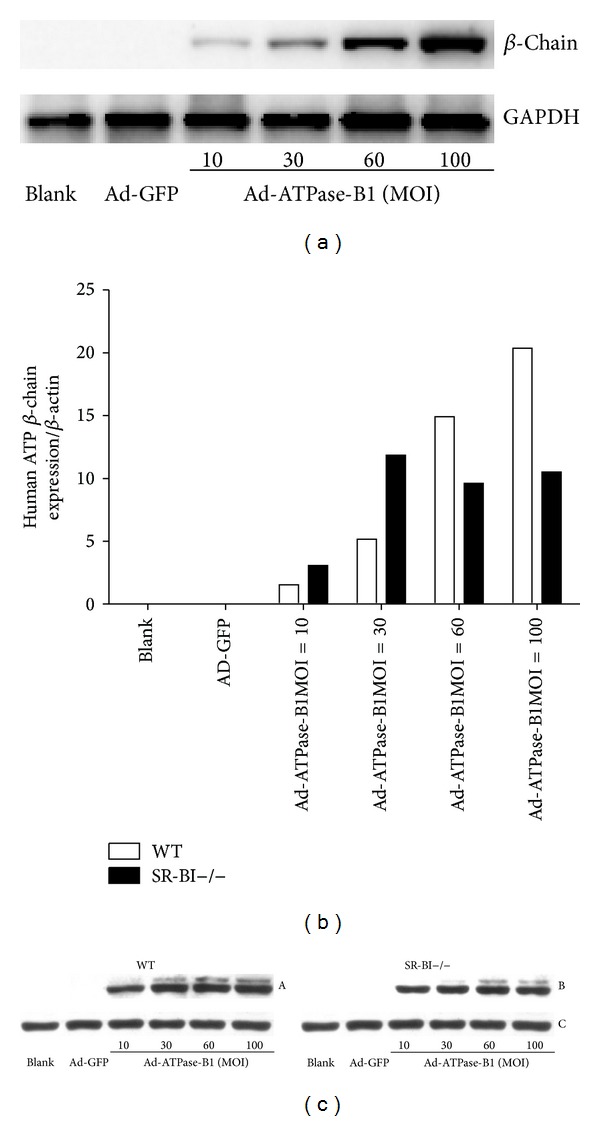
Multiplicity of infection (MOI) test in HepG2 cells and primary hepatocytes. HepG2 cells (a) and the isolated primary hepatocytes were pretreated with Ad-ATPase-B1 and Ad-GFP with different MOIs, and ATPase-B1 mRNA (b) and protein ((c) A, ATPase-B1 expression in WT primary hepatocytes; B, ATPase-B1 expression in SR-BI−/− primary hepatocytes; C, the internal control of GADPH in WT and SR-BI−/− primary hepatocytes) levels were measured. In HepG2 cells and WT hepatocytes, mRNA expression increased with increased MOI, while mRNA expression peaked at 30 MOIs in SR-BI−/− mouse.

To further investigate how different quantities of ATPase-B1 affect its cellular expression, we infected HepG2 cells with various concentrations of Ad-ATPase-B1 and Ad-GFP. Increased MOI corresponded to increased ATPase-B1 protein (Figure 3(a)) and mRNA expression (data not shown).

ATPase-B1 can be expressed on the surface of endothelial cells, adipocytes, hepatocytes, and tumor cells [24–28]. In order to determine MOI yielding the highest expression of Ad-ATPase-B1, we infected primary hepatocytes (WT and SR-BI−/− mice) with a range of different MOIs. While mRNA expression of Ad-ATPase-B1 was highest at 100 MOIs in the WT mice (Figure 3(b)), ATPase-B1 mRNA expression was highest at 30 MOIs in SR-B1−/− mice. Therefore, we used 30 MOIs Ad-ATPase-B1 for the remainder of our study. Unlike the HepG2 cells (Figure 3(a)), WT and SR-BI−/− hepatocytes showed slightly higher protein expression in response to higher MOIs (Figure 3(c))

### 2.3. ATPase-B1 Overexpression Increases DiI-HDL Uptake

**Figure 4:**
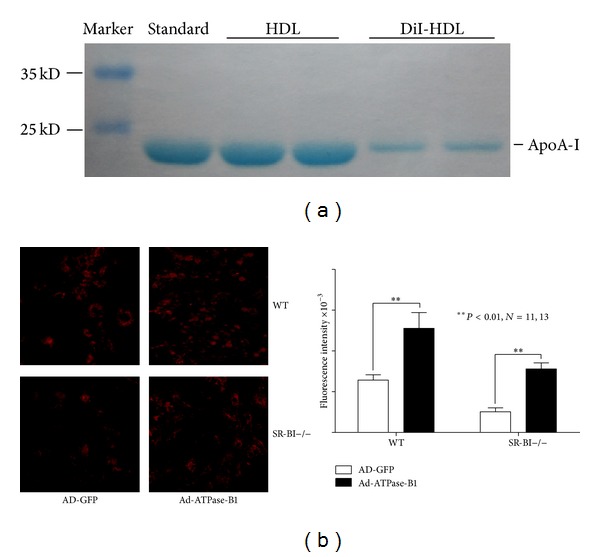
Analysis of DiI-labeled HDL uptake in freshly isolated primary hepatocytes (200x). Primary hepatocytes were pretreated with Ad-ATPase-B1 and Ad-GFP at 30 MOI for 48 h and incubated with 25 *μ*g DiI-HDL for 2 h. (a) Concentration of DiI-HDL and HDL was determined by the dying method with Coomassie brilliant blue. (b) DiI-HDL was detected by fluorescence microscopy (200x) and cellular fluorescent intensities were quantified.

We next investigated the effect of Ad-ATPase-B1 overexpression on DiI-HDL uptake. As shown in Figure 4(a), apoA-I (1 mg/mL) as the standard, the concentration of isolated HDL was 1 mg/mL, and DiI-HDL was 0.25 mg/mL. Freshly isolated primary hepatocytes were examined by fluorescence microscopy.

Figure 4(b) showed DiI-HDL uptake was significantly higher in Ad-ATPase-B1 infected WT and SR-BI−/− hepatocytes compared to their respective controls (*P* < 0.01). Although the DiI-HDL uptake is significantly higher in infected WT hepatocytes compared to the SR-BI−/− counterparts (*P* < 0.01), Ad-ATPase-B1 infection increased DiI-HDL uptake similarly in both groups (Table 1), suggesting that the lack of SR-BI does not affect ATPase-B1 function.

### 2.4. ATPase-B1 Overexpression Decreases Plasma HDL-C

**Figure 5:**
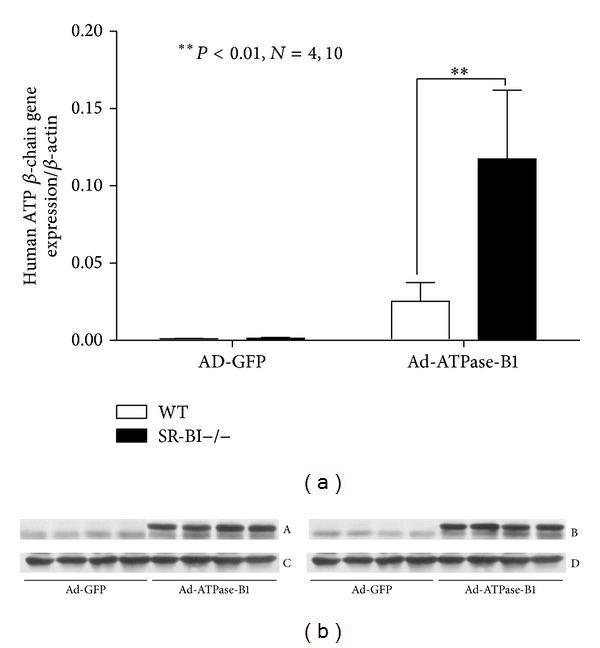
Verification of ATP-B1 expression in the livers of WT and SR-BI−/− mice by RT-PCR (a) and Western blotting ((b) A, ATPase-B1 expression in WT mouse liver; B, ATPase-B1 expression in SR-BI−/− mouse livers; C and D, the internal control of GADPH) after 7 days injection of Ad-ATPase-B1 and Ad-GFP. (**P < 0.001; *n* = 4, 10).

Ectopic ATPase-B1 has been characterized as an apoA-I receptor, triggering HDL internalization in hepatocytes [21]. To further assess the role of liver ATPase-B1 in HDL metabolism, we injected WT and SR-BI−/− (1 × 10^9^ pfu) mice with either Ad-ATPase-B1 or Ad-GFP via tail vein. Liver ATPase-B1 mRNA level increased significantly in both groups (Figure 5(a)); however, mRNA expression was significantly higher in the transfected SR-BI−/− mice (*P* < 0.001) compared to WT mouse. ATPase-B1 protein was detected in the livers of both WT and SR-BI−/− mice (Figure 5(b)).

**Figure 6:**
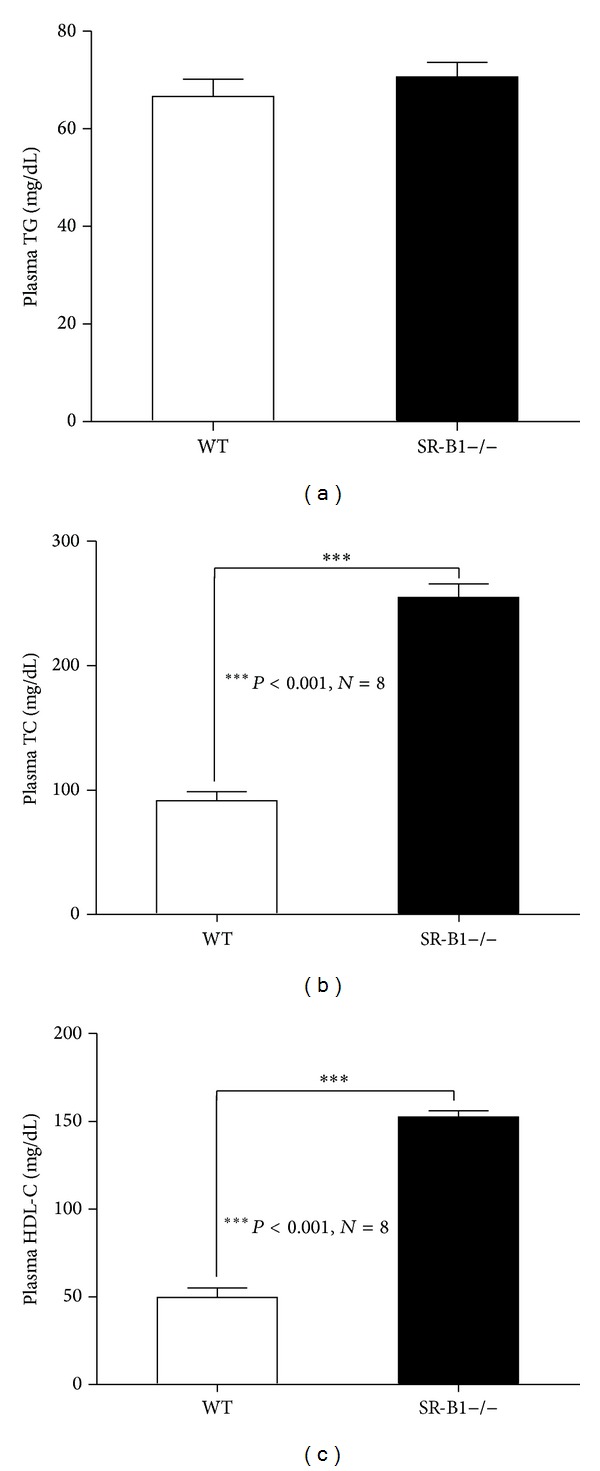
Plasma lipid levels in WT and SR-BI−/− mice. Before injection, plasma was collected after a 4 h fast. Plasma triglyceride (a), total cholesterol (b), and HDL-C (c) levels were detected.

**Figure 7:**
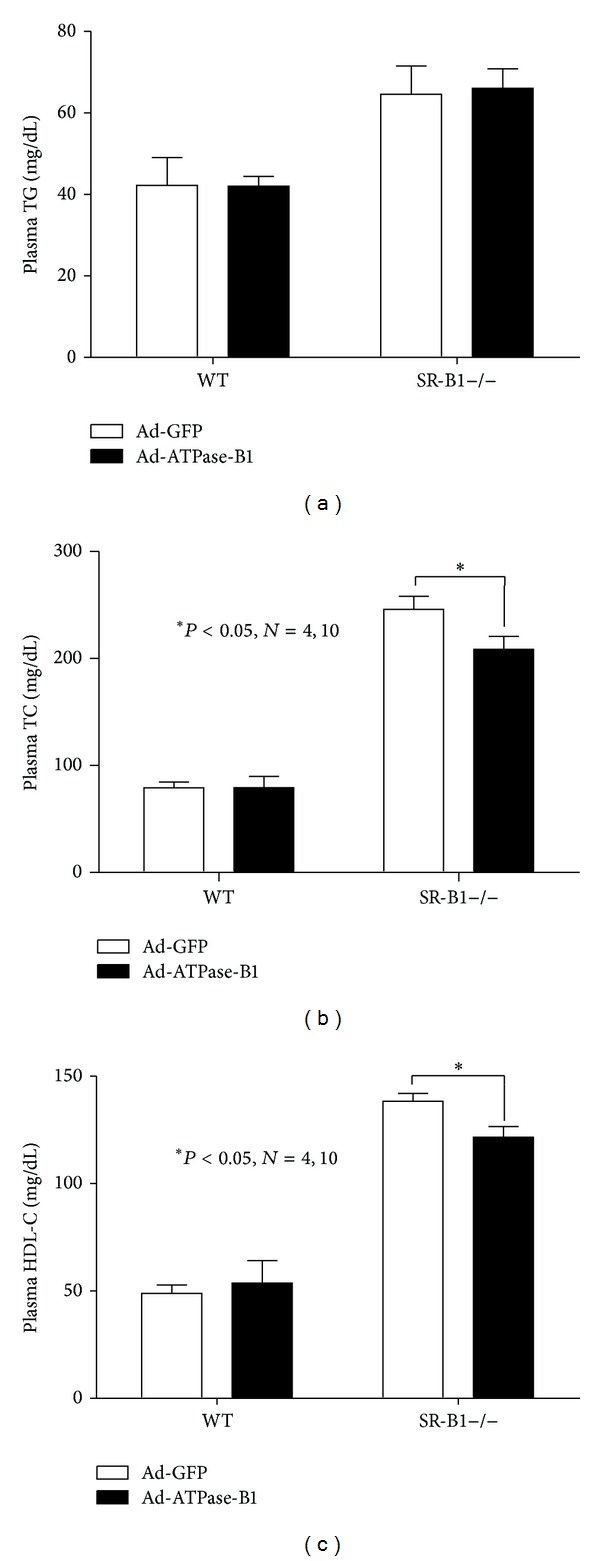
Plasma triglyceride (a), total cholesterol (b), and HDL-C (c) levels in WT and SR-BI−/− mice 7 days after administration of Ad-ATPase-B1 and Ad-GFP.

**Figure 8:**
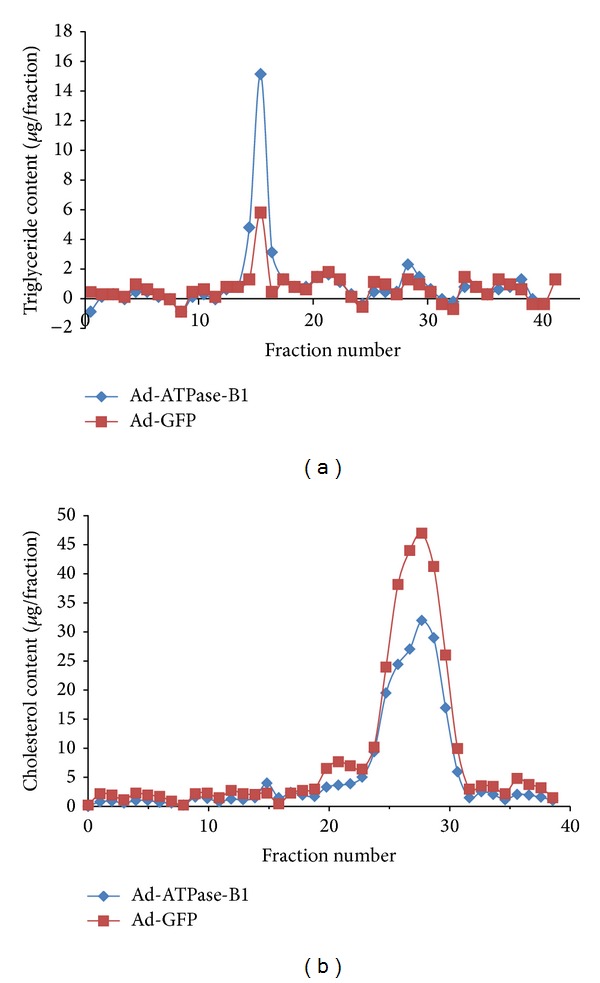
Plasma lipoprotein profiles. Seven days after Ad-ATPase-B1 and Ad-GFP administration, mice were fasted for 4 h and euthanized. Aliquots (250 *μ*L) of plasma pooled from each group of mice were fractionated by column gel filtration chromatography. Fractions (500 *μ*L) were eluted and assayed for TG (a) and cholesterol (b) levels.

Plasma total cholesterol and HDL-C were 2.5 and 3 times higher, respectively, in SR-BI−/− mice before infection (Figures 6(a)–6(c)). SR-BI−/− mice infected with Ad-ATPase-B1 showed lower plasma total cholesterol (~18%) and HDL-C (~12%) compared to SR-BI−/− mice infected with the control adenovirus (Figures 7(a)-7(b)), but plasma triglycerides were not different between the groups (Figure 7(c)). ATPase-B1 infection did not affect plasma total cholesterol, HDL-C, or triglycerides in WT mice (Figures 7(a)–7(c)). Analysis of lipoprotein profiles in the pooled plasma sample revealed lower HDL-cholesterol (Figure 8) in the Ad-ATPase-B1 mice compared to Ad-GFP mice. However, Ad-ATPase-B1 treated mice showed a significant increase in VLDL/CM-associated TG compared to Ad-GFP mice (Figure 8(a)).

## 3. Discussion

In this study, we set out to determine whether overexpression of ATPase-B1 affects plasma lipoprotein levels and whether this effect is mediated by SR-BI. ATPase-B1 is an enzyme located in the inner mitochondria membrane. A previous study showed that the surface *β*-chain is an apoA-I/HDL receptor [21], and the complex has been found on the cell surface of endothelial cells, adipocytes, hepatocytes, and tumor cells by immunofluorescence or after biotinylation of the cell surface [24–28]. Recently, new research has shown that knock-down of ABCA1, ABCG1, and SR-BI diminishes, but does not completely block, the transport of apoA-I or HDL through the endothelium [32, 33]. The ectopic presence of ATPase-B1 on the surface of endothelial cells was confirmed by cell surface biotinylation [31]. To investigate the effect of ATPase-B1 on HDL metabolism, we constructed an adenovirus containing the whole length of human ATPase-B1 and successfully transfected this Ad-ATPase-B1 into HEK293A cells (Figures 1(a) and 1(b)) and HepG2 cells (Figure 2(a)).

To further validate the role of ATPase-B1 on HDL metabolism, we measured DiI-HDL uptake after infecting primary hepatocytes with Ad-ATPase-B1. We demonstrate that overexpression of hepatic ATPase-B1 by adenovirus infection increases DiI-HDL uptake (Figure 4(b)) in cultured primary hepatocytes and decreases plasma total cholesterol and HDL-C (Figure 7) in SR-BI−/− mice. These findings agree with previous studies demonstrating that significant amounts of immunodetectable *β*-chain protein were present on the HepG2 cell surface and increased HDL uptake [29].

Although the infected WT hepatocytes had a greater absolute DiI-HDL uptake compared to the SR-BI−/− mice (*P* < 0.01), the difference in uptake between ATPase-B1 and GFP-infected mice was similar in both mouse models, suggesting that ATPase-B1 does not interact with SR-BI in increasing DiI-HDL uptake. Previous research in SR-BI−/− mice showed that plasma total cholesterol, HDL, and HDL volume were double that of a normal mouse [34, 35]. ATPase-B1 overexpression also decreased plasma total cholesterol (~18%) and HDL-C (~12%) (Figures 7(a)-7(b)) and showed a depletion of HDL-C lipoprotein profiles (Figure 8). However, our ATPase-B1 adenovirus is not specifically located in the cell membrane. Therefore, our infection procedure may not fully reflect the function of ATPase-B1 in HDL metabolism* in vivo*. Constructing an adenovirus specifically located in the cell membrane will help us understand the role of ATPase-B1 in HDL metabolism more clearly.

The exact mechanism of ATPase-B1 on the cell surface remains unclear. Previous research in hepatocytes shows that the *β*-chain functions as an apoA-I receptor and triggers HDL endocytosis [21]. Upon binding of apoA-I, ATP synthase hydrolyzes ATP into ADP and inorganic phosphate, and ADP stimulates hepatic HDL uptake by activating the purinergic receptor P2Y13 through the small GTPase RhoA and its effector ROCK I [28, 36]. However, whether this pathway is altered in SR-BI−/− mice is still unknown. Then, we will go through this pathway in future work.

Many genes participate in HDL and apoA-I metabolism, such as ATP-binding cassette transporter A1 (ABCA1) [37], ATP-binding cassette transporter G1 (ABCG1) [38–40], and SR-BI. However, whether ATPase-B1 interacts with them in HDL endocytosis is still unclear. Coinhibition experiments in endothelial cells suggest that ABCA1, ABCG1, SR-BI, and ATPase-B1 interact in a series of events rather than on independent parallel processes [31]. Future studies should aim to measure plasma apoA-I levels and determine whether there is an interaction among ATPase-B1, ABCA1, ABCG1, SR-BI, and apoA-I and the potentially downstream signaling pathway involved.

In summary, the present study demonstrated that overexpression of ATPase-B1 increased DiI-HDL uptake in primary hepatocytes and reduced plasma HDL-C and total cholesterol in SR-BI−/− mice and that ATPase-B1 increased HDL uptake independently of the presence of SR-BI. Future research should investigate the effect of ATPase-B1 on plasma apoAI and characterize the signaling events and downstream targets for HDL endocytosis.

## 4. Materials and Methods

### 4.1. Cell Culture

HepG2 and HEK293A cells were obtained from the American Type Culture Collection (ATCC, Manassas, VA) and maintained in DMEM containing 10% FBS (GIBCO, California, USA) supplemented with 100 U/mL penicillin G and 100 *μ*g/mL streptomycin sulfate at 37°C in a humidified atmosphere of 95% air, 5% CO_2_. For adenovirus infection experiments, cells were seeded in six-well plates at a density of 2 × 10^5^ cells per well and incubated in DMEM containing 5% FBS. Cells were infected with either Ad-ATPase-B1 or the control vector Ad-GFP.

### 4.2. Animal Studies

SR-BI−/− mice and WT mice were fed standard rodent chow and water* ad libitum* in sterile cages with a 12 h light/dark cycle. All mice used in this study were 12-week-old females. All the procedures involving mice were approved by Animal Ethics Committee. Two hundred microliters of a 0.9% sterile solution containing 1 × 10^9^ pfu of either Ad-ATPase-B1 or control Ad-GFP adenovirus was injected into the tail vein. Mice were fasted for 4 h and 200 *μ*L aliquots of orbital venous blood were collected after anesthesia into heparin-coated capillary tubes at 0 and 7 days. Plasma was collected by centrifuging samples at 4000 rpm for 10 min at 4°C and subsequently stored at −80°C. After 7 days, mice were anesthetized by intraperitoneal injection of pentobarbital, and tissue and blood were collected. Plasma levels of TG and cholesterol were determined using Thermo Infinity TG and cholesterol reagents (ThermoElectron, Melbourne, Australia). Plasma HDL-C levels were extracted using PEG precipitation and then determined using Thermo Infinity cholesterol reagents.

### 4.3. Recombinant Adenoviruses

ATPase-B1 cDNA was cloned into the plasmid pAd. The plasmid was linked to CMV using the restriction enzymes Bg*β* and Sph, with a V5-tag added to the C-terminal for detection purposes. CMV-*β*-chain was linearized with Nhe and cotransfected into HEK293A cells with Ad-DNA. The recombinant virus Ad-ATPase-B1 was plaque-purified, expanded, and purified on CsCl gradients as described by Kozarsky et al. [41]. The control adenovirus Ad-GFP was constructed using the same procedure but without the transgene expression cassette.

### 4.4. Primary Hepatocyte Isolation and Culture

Hepatocytes were isolated from WT and SR-BI−/− mice. The portal vein was cannulated, and the liver was perfused with KRG buffer (pH 7.4) containing 120 mM NaCl, 480 mM KCl, 120 mM MgSO_4_, 120 mM KH_2_PO_4_, and 50 mM EGTA. After perfusing at a rate of 1 mL/min at 37°C for 20 min, 50 mL collagenase buffer containing 1 mM CaCl and IV collagenase was added and perfusion continued for an additional 20 min. Cells were filtered to remove undigested fragments, centrifuged for 4 sec at 50 g, and washed twice in cold culture medium to remove damaged and nonliver cells. Isolated cells were seeded on six-well plates at a density of 3 × 10^5^ cells per well in DMEM medium containing 10% FBS. Culture medium was changed 4 h after seeding. The plated cells were infected with Ad-ATPase-B1 (titer: 8 × 10^10^ pfu/mL) in 1 mL of fresh DMEM at 37°C for 36 h at 30 multiplicities of infection (MOIs). One milliliter of complete medium was then added to each well. Parallel experiments were conducted using the control adenovirus vector (Ad-GFP, titer: 1 × 10^11^ pfu/mL) to infect the cells at the same MOI dose to determine the effect of adenovirus alone on the cells.

### 4.5. HDL Isolation and Labeling

Human HDL (1.063 < *d* < 1.21) was isolated from serum using density gradient centrifugation according to Redgrave et al. [42]. To remove KBr buffer, the isolated HDL was dialyzed with PBS containing EGTA-Na_2_ and stirred at 4°C for 48 h. The HDL was labeled with the fluorescence probe DiI (Beyotime) according to Pitas et al. [43] and was also dialyzed. HDL and DiI-HDL showed no differences in apoprotein composition on 12% SDS-PAGE [44].

### 4.6. HDL Uptake Assay

Primary hepatocyte uptake of DiI-HDL was analyzed using a fluorescence microscope. Briefly, the infected cells were cultured for 48 h and then incubated with 25 *μ*g DiI-HDL (0.25 mg/mL) at 37°C for 2 h in DMEM medium containing 5% FBS. Cells were then washed three times with PBS and fixed with 4% paraformaldehyde for 20 min at room temperature. Stained cells were imaged with fluorescence microscopy.

### 4.7. Western Blot Analysis

All cell and liver proteins were extracted following the standard protocol [45]. Proteins were separated on 10% SDS-PAGE gels and transferred onto nitrocellulose membranes. ATPase-B1 expression was analyzed using a mouse-anti-V5 monoclonal antibody (1 : 2000), primary antibody, and a goat-anti-mouse IgG-HRP (1 : 5000) secondary antibody. GAPDH (1 : 1000 dilution for the primary antibody and 1 : 5000 dilution for the secondary antibody) was used as an internal control.

### 4.8. RT and Quantitative Real-Time PCR

Total RNA extracted from cells and livers was reverse-transcribed using 10 units of M-MLV reverse transcriptase (Promega) following the standard procedure [46]. Real-time PCR was performed using QuantiTect SYBR Green PCR reagents (Life Technologies, California, CA). ATPase-B1 (TGGTGGTGCTGGAGTTGG, GCCTGGGTGAAGCGAAAG) transcription levels were normalized to *β*-actin (CGTGGGCCGCCCTAGGCACCA, TTGGCCTTAGGGTTCAGGGGGG).

### 4.9. Fast-Protein Liquid Chromatography Fractionation of Lipoproteins

Plasma aliquots (250 *μ*L) were pooled from a group of mice and applied to Tricorn high-performance Superose S-6 10/300 GL columns using a fast-protein liquid chromatography system (Amersham Biosciences), followed by elution with PBS at a constant flow rate of 0.25 mL/min. Eluted fractions (500 *μ*L) were assayed for TG and cholesterol concentrations using the TG and cholesterol kits (BioSino, China).

### 4.10. Confocal Microscopy

Freshly isolated primary hepatocytes grown on cover slips were pretreated with Ad-ATPase-B1 (A) and Ad-GFP (B) (MOI = 30) for 48 h. After three PBS washes, cells were fixed with 4% paraformaldehyde at room temperature for 5 min. After washing with PBS, the cells were immunostained with an antibody against V5-tag (Life Technologies, California, CA) (5 mg/mL) in PBS-1% BSA and AlexaFluor 596-conjugated goat anti-mouse IgG (5 mg/mL) (Molecular Probes) and analyzed using confocal microscopy (Leica SP5, Germany).

### 4.11. Statistics

Statistical analyses were performed using the PRISM statistics software. Analysis of variance (ANOVA) was used to compare the data. All experiments were performed in triplicate, and representative results are presented. Quantitative data are expressed as mean ± S.D. Student's *t*-test was used for statistical comparisons. *P* < 0.05 was considered statistically significant.

## Figures and Tables

**Table 1:** Mean fluorescence intensity in WT and SR-BI−/− primary hepatocytes.

Type/virus	Ad-GFP	Ad-B1
WT	3.5	6.8∗∗
SR-BI−/−	0.8	3.9∗∗

**Ad-B1 compared with Ad-GFP groups *P* < 0.01. The increment was 3.3 in WT group, while it was 3.1 in SR-BI−/− group; the difference had no statistical significance.

## Abbreviations

ATPase-B1: Adenovirus containing human holo-length ATP synthase *β*-chain
DiI-HDL: HDL was labeled with fluorescent dye (1,1′-dioctadecyl-3,3,3′,3′- tetramethylindocarbocyanine perchlorate)
HDL: High-density lipoprotein
SR-BI: Scavenger receptor class B, type I.
